# Alcohol and Drug Use in European University Health Science Students: Relationship with Self-Care Ability

**DOI:** 10.3390/ijerph16245042

**Published:** 2019-12-11

**Authors:** Natura Colomer-Pérez, Elena Chover-Sierra, Rut Navarro-Martínez, Virginia Andriusevičienė, Eugenia Vlachou, Omar Cauli

**Affiliations:** 1 Department of Nursing, University of Valencia, 46010 Valencia, Spain; natura.colomer@uv.es (N.C.-P.); elena.chover@uv.es (E.C.-S.); Rut.Navarro@uv.es (R.N.-M.); 2 Development and Advising in Traffic Safety (DATS) research group, INTRAS (Instituto de Investigación en Tráfico y Seguridad Vial), 46022 Valencia, Spain; 3 Hospital General Universitario, 46014 Valencia, Spain; evlachou@uniwa.gr; 4 Faculty of Health Care, University of Applied Sciences, 08303 Vilnius, Lithuania; ocauli@yahoo.com or; 5 Department of Nursing, School of Health and Caring Professions, University of West Attica, 12243 Athens, Greece

**Keywords:** Appraisal of Self-care Agency, substance abuse, alcohol consumption, AUDIT, CRAFFT, university students

## Abstract

*Background*: Drug abuse in university students is an emerging social and health issue. The present study assesses alcohol and abuse of other illicit drugs and the adverse consequences related to such use and its relationship with self-care agency among European university students. *Methods*: A multicenter cross-sectional study was performed among 592 European students from different health science degrees. The screening of alcohol abuse was evaluated with the Alcohol Use Disorder Test (AUDIT), and the screening for substance-related risks and problems was conducted with the Car, Relax, Alone, Forget, Friends, Trouble (CRAFFT) screening test. We analyzed the relationship of substance abuse with self-care agency, assessed by the Appraisal of Self-Care Agency Scale (ASA). *Results*: 51.4% of the surveyed students reported alcohol intake, 16.6% of the students consumed both alcohol and cannabis, 1.6 % reported alcohol and other illicit drugs, and 3.7% consumed alcohol, marijuana, and other illicit drugs (73.3% of students reported alcohol intake alone or together with cannabis/hashish and/or other illicit drugs). The self-care agency scores were significantly different among groups in relation to certain sociodemographic factors such as gender (*p* = 0.008) and country of residence (*p* = 0.031). The self-care agency scores significantly correlated (*p* = 0.001) with the personal motivations and consequences related to the consumption of drugs of abuse evaluated by the CRAFFT screening tool. Within the ASA domains, the most significant effects were observed regarding the subdomains of resources, awareness, and health behaviors. *Conclusions*: Self-care agency should be promoted to counteract the health and social consequences of the consumption of drugs of abuse among university students who will be future health care professionals.

## 1. Introduction

In recent decades, health agencies and university authorities have expressed concerns over increasing alcohol consumption and also other drugs of abuse such as cannabis and amphetamines among university students [1,2,3,4,5]. Moreover, alcohol often plays an essential role in young people’s lives when they enter university [6]. Multiple factors contribute to young university student risk-related alcohol use [7,8], though in particular, university students are at risk of substance abuse behaviors due to changes in lifestyle, reduced parental support, and the presence of stressful situations [9]. Higher education studies offered by universities and specifically in the health sciences should provide knowledge about the harmful health and social consequences of the use and abuse of alcohol and other drugs, which represent significant health and social concern in university life [10]. The knowledge of the toxic and social effects of drug abuse are well known by university students; however, the ability to self-care for proper and good health may vary among people. In this respect, the self-care concept represents the practice of daily activities for maintaining life, health, and well-being [11]. The ability to exercise self-care actions begins to develop in childhood, generally acquires its peak in adulthood, and begins to decline when advancing in age. Such human ability lies at the basis of the theory framed within the general theory of self-care, which, besides self-care, includes “self-care agency,” defined as the ability to engage in self-care, conditioned by age, developmental state, life experience, sociocultural orientation, health, and available resources [11]. The self-care agency of each person is influenced by intellectual curiosity, the formation and supervision of external agents, and by the contrast with their own experience through which self-care measures are taken. Different instruments have been validated to measure self-care agency, including the Appraisal of Self-Care Agency (ASA) [12,13,14,15,16,17], and they have been applied in different pathogenic situations such as heart and kidney diseases, diabetes, mental health disorders, hepatitis, and several other circumstances [16,18,19,20].

The realization of self-care requires both intention and action and is conditioned by the knowledge and repertoire of skills and education of the individual. In this regard, university students, and in particular, those studying health sciences, should be the best candidates to acquire self-care abilities and behave in a way that would limit the adverse health consequences of inadequate lifestyles [21,22,23]. However, university students report exciting and empowering experiences throughout their university lives, including important lifestyle changes (leaving the family home to live alone or with flatmates, moving to other cities, seeking independence in the making of life decisions, wishing to earn their own money, etc.), and this may influence alcohol and drug abuse consumption. In addition, university students endure stress due to the academic workload, pressure to succeed, and competition among peers.

The main objective was to assess self-care knowledge with the ASA in university health science students and its relationship with the consumption of alcohol and drug abuse.

## 2. Materials and Methods

### 2.1. Study Design and Sample

A cross-sectional design study was conducted, recruiting university students from different European universities. The inclusion criteria were the following: Students enrolled in a degree in health sciences at the participating European universities during the academic year of 2017–2018. This multicenter study arose from the European contact network partnership of this research group, which was consolidated through Professor Erasmus Mobility projects over the last three years. The survey fulfills the criteria of the Declaration of Helsinki (2000). The Ethics Committee of the University of Valencia was consulted to assess the suitability of the research and they approved the research design (protocol H1480590883286, dated 21 December 2016). The study was conducted guaranteeing the anonymity of its participants, with emphasis on the existing laws referring to data protection and the fact that the information would only be used for statistical and research purposes. For this purpose, previous permission from the academic institutions had to be agreed on with the cooperating institutions. Students were emailed a web link sent by their university’s academic authority or through social networks in order to access an anonymous and self-administered questionnaire, designed ad hoc with Google Forms. The participants were recruited between September 2017 and January 2018. Several reminders were sent during this period by local academic authorities and social media. The questionnaire was in English and included information about sociodemographic data, such as the student’s age, gender, nationality, degree they were studying, years studying at university, their employment situation, housing situation/place of residence, whether they had children, and whether they suffered from a chronic disease. The participants also completed a questionnaire package consisting of validated self-rating instruments about self-care ability, alcohol consumption, and substance abuse, as explained below.

### 2.2. Evaluation of Substances Abuse

The risk of abusive alcohol consumption was assessed using the Alcohol Use Disorders Identification Test (AUDIT test) [24]. This is a simple screening method developed by the World Health Organization (WHO) to identify a pattern of risky or harmful alcohol consumption that has demonstrated reasonable psychometric performance in university students [25]. This 10-item scale evaluates three conceptual domains: Hazardous alcohol use (items 1–3), dependence symptoms (items 4–6), and harmful alcohol use (items 7–10). To assess substance abuse (alcohol, cannabis, and “anything else,” including illegal, over-the-counter, and prescription drugs) and its consequences, we used the Car, Relax, Alone, Forget, Friends, Trouble (CRAFFT) screening tool, [26] a brief and effective screening device comprising a series of 6 questions developed to screen young people for high-risk alcohol and other drug use disorders simultaneously. The name of the tool is a mnemonic acronym of the first letters of keywords in the six screening questions used to evaluate the consequences, e.g., Car, Relax, Alone, Forget, Friends, and Trouble. The CRAFFT consists of two parts: A first screening, part A, composed of three questions referring to alcohol, marijuana, and other drugs consumed in the last 12 months; and a second part B comprising six questions about problems related to the consumption of such substances. The response format is dichotomous (yes/no). If the answers to the three questions from part A are “No”, only the first question from part B of the questionnaire is asked. In contrast, if “Yes” is answered to any of the three items of part A, part B of the scale is carried out, which is the only part where the score is given. In the case of a negative response (no), a score of zero is assigned, while an affirmative answer (yes) is assigned a score of one point. To evaluate the instrument, the scores of the six items of part B are added up. Scores equal to or greater than 2 suggest the presence of abusive consumption [27].

### 2.3. Evaluation of the Appraisal of Self-Care Agency (ASA)

The Appraisal of Self-care Agency Scale (ASA-S) was measured based on a 24-item scale in which each item was scored on a 5-point Likert scale. The scale included questions related to perceived health self-care. Thus, the ASA-S construct reflects five dimensions [28]: “Resources”, with 9 items (9, 10, 12, 16, 17, 18, 19, 21, 22) and a scope score of 9–45 points; “Ignorance”, with 6 items (2, 6, 11, 13, 20, 23) and a scope score of 6–30 points; “Ability” with 4 items (6, 10, 21, 24) and a scope score of 4–20 points; “Health behavior”, with 7 items (1, 3, 4, 5, 7, 8, 21) and a scope score of 7–35 points; and “Health awareness”, with 3 items (14, 15, 24) and a scope score of 3–15 points. The total sum ranged from 24 to 120. The sum of the scores in the 5 subdomains was not equal to the total ASA score, because in some subdomains, some of the items were repeated. Higher scores indicated better capability to take care of personal health and procure well-being for oneself.

### 2.4. Statistical Analyses

Descriptive statistics (frequency, mean, range, and standard error of the mean (SEM)) were conducted for sociodemographic variables and for the ASA, AUDIT, and CRAFFT scores. After confirming the non-normal distribution of quantitative variables with the Shapiro–Wilk, which was used to evaluate the skewness of a distribution, non-parametric statistical tools were used: The Mann–Whitney U-test and the Kruskal–Wallis H test for exploring differences between groups, and the Spearman test for correlation analysis between quantitative variables. We estimated the effect size by calculating Cohen’s d (calculated from Eta squared) or Hedges’ g for group comparison as a measurement of effect size when the *p* values were significant (*p* < 0.05). Hedges’ g provided a measure of effect size, weighted according to the relative size of each sample, and an alternative where there were different sample sizes. The number of potential confounders and the level of their grouping was taken into account by applying multivariate analysis, which represented a valid solution to control confounding factors with multivariate analysis of covariance (MANCOVA) analysis [29]. Specifically, in order to examine the role of sociodemographic variables in alcohol and drug abuse (risky or not risky behaviors based on AUDIT and CRAFFT cut-off scores, respectively), we devised a logistic regression analysis model that included the variables associated with risky use in the bivariate analysis. Statistical significance was considered for *p* < 0.05. The Statistical Package for the Social Sciences (SPSS), version 24.0 (Armonk, NY: IBM Corp. Armonk, New York, NY, USA).

## 3. Results

### 3.1. Characteristics of the Study Sample

The population of students from the European contact network partnership of this research group was 2690. The calculation of the sample size resulted in at least 340 subjects being randomly selected from the population in order to estimate with a 95% confidence and a precision ± 1 units. A population mean of values that were considered presented a standard deviation of 9 units (based on CRAFFT score range). A replacement rate of 20% was anticipated because the nature of the study of “drug abuse” could have losses, even if it was anonymous for all participants. The descriptive analysis of the sample referring to the sociodemographic data is reported in Table 1.

A total of 592 university students (484 women and 108 men) were finally included in the study. Their ages ranged from 17 to 26 years, with a mean age of 20.45 ± 0.08 years (CI 95%: 20.30–20.60), and the majority were Spanish (*n* = 289). More than half of the sample (75.7%; *n* = 448) were nursing students, 7.3% were medical students (*n* = 43), and 1.7% were pharmacy students (*n* = 10). The rest of the subjects were attending courses in psychology, nutrition, physiotherapy, odontology, and some other related disciplines of health science degrees, and only 9.8% of them (*n* = 58) reported having previously studied another degree in the health sciences. Regardless of the degree they were studying, 30.6% of the subjects (*n* = 181) had been studying at the university for one year, 19.8% for two years (*n* = 117), 15.2% for three years (*n* = 90), 23.8% for four years (*n* = 141), 5.7% for 5 years (*n* = 34), and 4.9% for 6 or more years (*n* = 29). We likewise documented certain parameters such as the co-living unit reported by the students. This study variable confirmed that 68.1% of the subjects (*n* = 403) were living with their parents or other relatives (ascendant relatives), 3.5% (*n* = 21) were living with a partner or children (descendent relatives), 21.1% (*n* = 125) with flatmates, and only 7.3% (*n* = 43) lived alone. Four students reported having children.

### 3.2. Evaluation of Health Self-Care

The mean score of the ASA construct was 82.28 ± 0.38 (C.I. 95%: 81.55–83.02). For this sample, the range of this construct was 47–109. The mean ± SEM values (CI 95%) of the ASA subdomains were 33.35 ± 0.21 (32.94–33.77) for “resources”, 16.86 ± 0.15 (16.56–17.15) for “ignorance”, 12.95 ± 0.09 (12.77–13.14) for “ability”, 27.91 ± 0.16 (27.60–28.22) for “health behavior” and 12.01 ± 0.10 (11.82–12.21) for “health awareness” (Table 2). The sum of the scores in the 5 subdomains is not equal to the total ASA score because, in some subdomains, some of the items are repeated (see Methods section). A significant gender difference (*p* = 0.008, Mann–Whitney U-test) was found in ASA-S scores, where females yielded significantly lower scores than males (Hedges’ g = −0.286).

Table 3 shows ASA-S and its subdomains’ scores according to sociodemographic variables. Significant differences in total ASA-S scores were also observed among countries: Italian students showed higher ASA-S scores (85.62 ± 1.29), while Lithuanian students showed lower scores (79.60 ± 1.05) (*p* = 0.008, Kruskal–Wallis test) (Cohen’s d = 0.26). Among the ASA-S subdomains, Lithuanian students reported significantly higher scores in the “Health Behavior” domain (*p* = 0.002, Kruskal–Wallis test) (Cohen’s d = 0.414). In addition, students who had an employer scored significantly lower in ASA-S than those without a job (*p* = 0.042, Mann–Whitney U-test) with a small effect size (Hedges’ g = 0.184). No significant differences were found regarding the coliving units, the degree they studied, or the presence of chronic diseases in relation to the ASA scores.

### 3.3. Evaluation of Substance Abuse

The mean AUDIT score referring to “alcohol use disorders” was 3.14 ± 0.10 (CI 95%: 2.94–3.35); 6.9% of the students were considered hazardous drinkers (AUDIT > 8) based on the risk categories proposed by the WHO (Table 2). Regarding the CRAFFT score, 75.8% (*n* = 449) of the students answered “Yes” to at least one of the questions of part A, and therefore answered all the questions of part B of the CRAFFT scale. Then, 73.3% (*n* = 434) reported having consumed alcohol in the last year alone or alcohol with cannabis/hashish and/or other illicit drugs), 22.1% (*n* = 131) marijuana or hashish, and 6.8% (*n* = 40) other substances. Likewise, while 51.4% (*n* = 304) of the students reported having consumed alcohol exclusively, 16.6% (*n* = 98) of them consumed both alcohol and marijuana, 1.7% (*n* = 10) alcohol and drugs other than marijuana, and 3.7% (*n* = 22) alcohol, marijuana, and other drugs. When selecting the cut-off of score 2 for the CRAFFT screening tool, 354 (59.8% of the sample) fulfilled the criteria for risky consumption. Several significant differences were found in the CRAFFT score based on sociodemographic data. Students who reported a co-living unit shared with flatmates had the highest global CRAFFT scores (2.76 ± 0.16 (CI 95%: 2.44–3.08), *p* = 0.002; Kruskal–Wallis test) (Cohen’s d = 0.281). Lithuanian students scored significantly higher in the global CRAFFT score than students from other countries (2.44 ± 0.24 (CI 95%: 1.97–2.91), *p* = 0.01; Kruskal–Wallis test) (Cohen’s d = 0.490). Nursing students had lower scores in the CRAFFT part B score than students from other health sciences (1.20 ± 0.06 (CI 95%: 1.07–1.32), *p* = 0.03; Mann–Whitney U test); however, the difference had a limited effect size (Hedges’ g = −0.177). Finally, students who had a job scored significantly higher in the global CRAFFT score (2.59 ± 0.15 (CI 95%: 2.29–2.88), *p* = 0.01; Mann–Whitney U test) (Hedges’ g = 0.305). Finally, an odds ratio (OR) analysis showed that working during university studies was significantly associated (*p* = 0.029) with problematic drug use as expressed by the CRAFFT score, with an OR = 1.502 (CI 95%: 1.041–2.167). OR analysis showed that living with flatmates or alone during university studies was significantly associated (*p* = 0.001) with problematic drug use as expressed by the CRAFFT score, with an OR = 2.030 (CI 95%: 1.380–2.985). Logistic regression analysis showed that when all the sociodemographic variables that had a significant relationship with the CRAFFT score were added to the model (country, type of university degree, employment situation, and coliving modality during studying), the risky consumption based on CRAFFT score (≥2) was significantly associated with coliving status (living with flatmates) (*p* = 0.002, Exp (B) = 1.929, CI 95%: 1.276–2.917) and with having had an employer (*p* = 0.001, Exp (B) =1.971, CI 95%: 1.301–2.987) during university studies. On the other hand, the only significant correlation between AUDIT and age was very small (rho = −0.082 and *p* = 0.045), and when we dichotomized students based on AUDIT score as having or not having risky consumption (AUDIT score ≥8 or <8, respectively), we did not observe any significant difference, and logistic regression analysis was not significant either for the variable “age”.

### 3.4. Correlation Analysis between Health Self-Care and Substance Abuse

**Figure 1:**
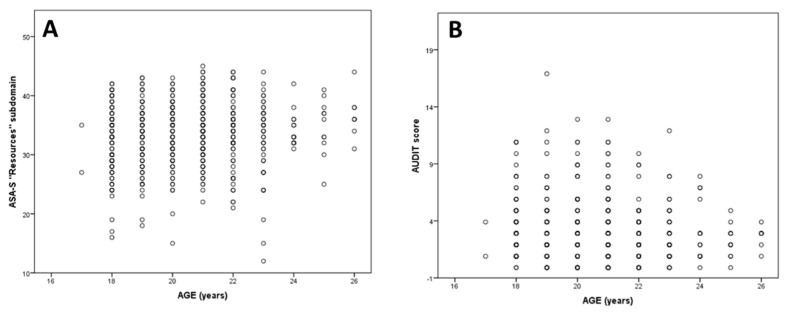
Significant correlations between age variable with Appraisal of Self-care Agency Scale (ASA-S) (**A**)and Alcohol Use Disorder Test (AUDIT) (**B**) instruments.

Some important correlations were observed between sociodemographic variables. A significant association between ASA-S’s “Resources” dimension and age was found (rho = 0.112, *p* = 0.007; Spearman test), meaning that the older the age, the greater the reported health self-care (Figure 1A). AUDIT global scores showed a slightly significant relationship with the results in ASA’s Ability component (rho = 0.083, *p* = 0.043; Spearman test) and with age (rho = −0.082, *p* = 0.045; Spearman test) (Figure 1B).

**Figure 2:**
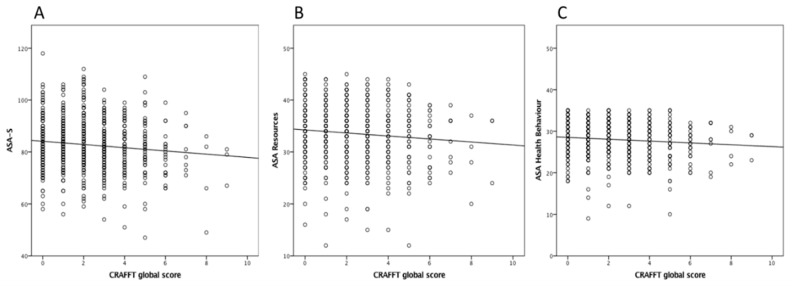
Significant correlations between CRAFFT and ASA-S (**A**), ASA-Resources (**B**) and ASA Health Behaviour (**C**).

The AUDIT “Hazardous alcohol us” inner dimension also showed a significant indirect correlation with age (rho = −0.098, *p* = 0.018; Spearman test). Moreover, significant associations were found between the AUDIT’s scores and the subdomains “Hazardous alcohol use” (rho = 0.897, *p* = 0.000; Spearman test). These correlations remained significant (*p* < 0.05) even after adjusting for the confounding variables, gender and nationality. No significant correlations were found between the global scores of both instruments (ASA-S and AUDIT) or between both inner domains. On analyzing the correlations between reported self-care agency and the presence of problems with substance consumption as measured with the CRAFFT instrument (part B), a significant inverse correlation was found (rho = −0.106, p = 0.01; Spearman test) (Figure 2A).

The evidence showed that the higher the reported health self-care agency score, the lower the presence of abusive substance consumption. Likewise, similar significant contrasting correlations with these results in CRAFFT part B were found for “Resources” (rho = −0.083, *p* = 0.044; Spearman test) and “Health Behaviour” (rho = −0.093, *p* = 0.024; Spearman test) subdimensions of the ASA-S (Figure 2B,C).

## 4. Discussion

The results obtained show two novel findings: Evaluation of drug abuse among university students in health sciences, the factors affecting the capability of self-care agency in a sample of European university students, and their relationships.

These data support the following main ideas: (i) A relevant percentage of university students fulfilled criteria for risky drug abuse; (ii) the extent of self-care agency (ASA) depends on certain sociodemographic factors; (iii) self-care agency is associated with risky consumption of drugs of abuse and its consequences. These ideas are discussed below.

A percentage as high as 73.3% of the sample reported having consumed alcohol in the last year, 22.1% marijuana or hashish, and 6.8% other illicit drugs. According to the risk categories proposed by the WHO, 6.9% of the students can be considered hazardous drinkers (AUDIT ≥8). This prevalence is high for adult students belonging to higher educational levels, and moreover studying university degrees in health sciences, and supports the findings of recent studies developed among university students regarding the use of alcohol [30,31,32,33], cannabis [31,33,34], and other illicit drugs [31,32,33,34,35]. A study performed among Mexican university students suggested different factors associated with changes in role and status, friendship, and increased autonomy as reasons for alcohol use after entering university [32]. There were significant differences in the CRAFFT screening tool depending on the sociodemographic variables. Northern countries such as Lithuania presented higher scores, confirming previous studies performed in Europe at the level of the general population [36]. Studying nursing is associated with a lower score in the CRAFFT tool, which fits well with learning to avoid unhealthy habits as a cornerstone in the care of individuals. The latter effects have limited effect size in this study, and future research needs to be designed in order to specifically address the influence of living country and the type of university study on the risk of consequences from consuming drugs of abuse. In contrast, logistic regression analysis shows that living with family is a protective factor, and working during university studies is detrimental for risky consumption compared to living with flatmates or alone, supporting previous studies performed with high school students [37]. Interventions to counter such risky behaviors should involve, in addition, strengthening prosocial involvement and parental monitoring.

The ability of self-care agency differed significantly between genders and countries. The ASA score was higher in males compared to females, and although the effect was small, it was statistically significant. This is a relevant finding considering that most of the students in the study sample were females studying nursing (i.e., future healthcare professionals), and that the opposite was expected to be found. It is therefore imperative for nursing programs to adequately prepare students for the responsibility of patient care. Regarding the components of the ASA score, the majority of them were higher in males than in females, thus suggesting that male students might have greater knowledge and practice healthy habits. In accordance with these findings, other recent studies have shown overweight and sedentary lifestyles to be more prevalent among female students compared to male students [38,39]. To date, no studies have analyzed gender differences of self-care agency in university students, though several studies conducted in adults support our observations. For instance, among patients with heart failure, a higher perceived control and better knowledge were related to better self-care behaviors in men, while higher self-care confidence and poorer functional status were related to better self-care behaviors in women [40]. Male patients undergoing hemodialysis showed a higher mean overall self-care agency score than women [41]. Women with hearth failure and other cardiac conditions are more likely to suffer psychosocial distress and need more social support than men [42], and both psychological distress and lower social support have been related to poor self-care in several studies [43,44,45]. In a study of adolescents with type 1 diabetes mellitus, female adolescents had weaker self-care performance in comparison with male subjects [46]. ASA-score as well as CRAFFT score showed differences among university students from different European countries, e.g., higher in Italian and lower in Lithuanian students, which could be partially due to different educational, cultural, and social aspects in different European countries when comparing northern and southern countries.

The ASA score was not associated with the use of alcohol, cannabis, or other illicit drugs (cocaine, amphetamines, etc.) (AUDIT or part A of the CRAFFT scale), but rather with the social and personal consequences derived from drug abuse (part B and total CRAFFT scores). It investigated whether the adolescent had used the substance, and represented a measurement of drug-related problems [26]. Further studies are needed to characterize these associations to establish educational and health interventions to counteract the personal events related to habits of drug abuse in students. Among the ASA subdomains, those significantly associated with drug abuse were “Resources” and “Health behavior”, suggesting that educational strategies should focus on expanding student knowledge about the health consequences of drug abuse and promote behaviors that encourage healthy living habits such as sports, physical activity, and outdoor leisure time activities. Based on this approach, perceived health self-care should be treated from a comprehensive perspective, also considering the psychological, cultural, and social aspects.

Educational intervention campaigns could be a strategy for dealing with the problem, employing a set of strategies that have been shown to be effective in changing alcohol-related behaviors into healthier ones [47]. Confirming the efficacy of these types of interventions, a clinical trial in the United Kingdom implemented a health self-care behavioral intervention for new university students consisting of self-affirmation manipulation, health messages based on the theory of planned behavior, and the implementation of intention tasks, pursuing lower abuse of alcohol [48].

## 5. Conclusions

There is an important issue regarding unhealthy life style habits of University students in health sciences related to the exposure of substances of abuse like alcohol, cannabis derivatives and other illicitis drugs. Such exposure is associated with adverse outcomes concerning several social and personal issues. The self-care agency appears to be involved in these adverse outcomes and should be promoted to counteract the health and social consequences of the consumption of drugs of abuse among university students who will be future health care professionals.

## Figures and Tables

**Table 1:** Sociodemographic variables of the sample.

Characteristics		Mean (SEM)	Range
Age (years)		20.45 (0.08)	17–26
Years at the university		2.70 (0.06)	1–7
		*n*	%
Gender	Female	484	81.8
	Male	108	18.2
Nationality	Spanish	289	48.8
	Italian	47	7.9
	Greek	109	18.4
	Lithuanian	50	8.4
	Others	57	9.6
University degree	Nursing	448	75.7
	Others	144	24.3
Previous studies	Yes	58	9.8
	No	534	90.2
Co-living unit	Ascendant relatives	403	68.1
	Descendent relatives	21	3.5
	Friends/Flatmates	125	21.1
	Alone	43	7.3
Children	Yes	4	0.7
	No	588	99.3
Employment situation	Working	179	30.2
	Not working	413	69.8
Chronic disease	Yes	88	14.9
	No	504	85.1
English level	A1	33	5.6
	A2	78	13.2
	B1	167	28.2
	B2	198	33.4
	C1	90	15.2
	C2	26	4.4

**Table 2:** Evaluation of self-care agency, alcohol, and drug abuse. ASA-S: Appraisal of Self-care Agency Scale; AUDIT: Alcohol Use Disorders Identification Test; CRAFFT: Car, Relax, Alone, Forget, Friends, Trouble screening tool.

	Mean (SEM)	Minimum	Maximum
ASA-S	82.28 (0.38)	47	109
Resources	33.35 (0.21)	12	45
Ignorance	16.86 (0.15)	6	30
Ability	12.95 (0.09)	6	20
Health Behaviour	27.91 (0.16)	9	35
Health Awareness	12.01 (0.10)	5	20
AUDIT	3.14 (0.10)	0	17
Hazardous alcohol use	2.69 (0.08)	0	10
Harmful alcohol use	1.15 (0.08)	0	14
Dependence symptoms	0.47 (0.05)	0	10
CRAFFT A	1.02 (0.31)	0	6
CRAFFT B	1.26 (0.06)	0	3
CRAFFT global	2.27 (0.08)	0	9

**Table 3:** Analysis of Appraisal of Self-care Agency Scale (ASA-S) and dimensions based on sociodemographic factors.

Variable		ASA-S	*p*	Resources	*p*	Ignorance	*p*	Ability	*p*	Health Behavior	*p*	Health Awareness	*p*
		Mean (SEM) (CI 95%)		Mean (SEM) (CI 95%)		Mean (SEM) (CI 95%)		Mean ± SEM (CI 95%)		Mean (SEM) (CI 95%)		Mean (SEM) (CI 95%)	
Gender	Male	84.24 (0.78) (82.69–85.79)	0.008	34.19 (0.44) (33.32–35.05)	0.084	17.31 (0.36) (16.60–18.03)	0.101	12.84 (0.19) (12.46–13.23)	0.674	28.47 (0.34) (27.81–29.14)	0.140	12.31 (0.24) (11.83–12.80)	0.209
	Female	81.85 (0.42) (81.02–82.68)		33.17 (0.24) (32.70–33.64)		16.75 (0.16) (16.43–17.08)		12.98 (0.11) (12.77–13.19)		27.78 (0.18) (27.43–28.13)		11.95 (0.11) (11.73–12.16)	
Nationality	Spanish	82.49 (0.55) (81.41–83.57)	0.031	33.42 (0.30) (32.83–34.00)	0.260	16.92 (0.21) (16.50–17.34)	0.061	12.87 (0.13) (12.62–13.12)	0.086	28.11 (0.22) (27.68–28.54)	0.002	11.87 (0.15) (11.57–12.17)	0.062
	Italian	85.62 (1.29) (83.02–88.21)		34.40 (0.78) (32.84–35.97)		17.45 (0.60) (16.23–18.66)		12.68 (0.31) (12.06–13.30)		29.06 (0.47) (28.12–30)		12.72 (0.36) (12.00–13.44)	
	Greek	82.58 (0.97) (80.66–84.50)		33.29 (0.52) (32.26–34.33)		17.22 (0.36) (16.52–17.92)		12.96 (0.23) (12.51–13.42)		27.59 (0.41) (26.79–28.39)		12.35 (0.23) (11.89–12.80)	
	Belgian	80.58 (1.08) (78.40–82.75)		32.18 (0.71) (30.74–33.61)		17.10 (0.46) (16.16–18.04)		12.50 (0.39) (11.70–13.30)		26.15 (0.65) (24.83–27.47)		12.38 (0.25) (11.87–12.88)	
	Lithuanian	79.60 (1.05) (77.49–81.71)		32.66 (0.69) (31.27–34.05)		16.32 (0.51) (15.29–17.35)		13.80 (0.33) (13.14–14.46)		26.84 (0.56) (25.72–27.96)		11.60 (0.29) (11.01–12.19)	
	Others	81.49 (1.13) (79.22–83.76)		33.72 (0.68) (32.36–35.08)		15.63 (0.44) (14.76–16.51)		13.14 (0.32) (12.51–13.78)		28.70 (0.46) (27.78–29.62)		11.61 (0.32) (10.97–12.26)	
University degree	Nursing	82.13 (0.42) (81.30–82.96)	0.653	33.25 (0.24) (32.77–33.72)	0.493	16.82 (0.17) (16.48–17.15)	0.650	12.92 (0.11) (12.71–13.14)	0.657	27.91 (0.18) (27.56–28.26)	0.943	12.01 (0.12) (11.78–12.23)	0.715
	Others	82.76 (0.81) (81.17–84.35)		33.69 (0.43) (32.85–34.53)		16.98 (0.31) (16.38–17.58)		13.05 (0.18) (12.69–13.41)		27.89 (0.34) (27.21–28.57)		12.03 (0.21) (11.62–12.44)	
Employment situation	Not working	82.82 (0.46) (81.92–83.71)	0.042	33.49 (0.25) (32.99–33.98)	0.176	17.08 (0.18) (16.73–17.43)	0.065	12.89 (0.11) (12.68–13.11)	0.388	28.06 (0.18) (27.70–28.42)	0.789	12.01 (0.12) (11.77–12.24)	0.497
	Working	81.05 ± 0.66 (79.75–82.35)		33.05 ± 0.39 (32.29–33.81)		16.34 ±0.28 (15.80–16.88)		13.09 ±0.18 (12.73–13.45)		27.55 ± 0.30 (26.95–28.14)		12.03 ± 0.18 (11.67–12.39)	
Chronic disease	Yes	83.17 (1.08) (81.02–85.32)	0.203	33.85 (0.56) (32.73–34.97)	0.384	17.06 (0.38) (16.30–17.81)	0.651	12.95 (0.23) (12.50–13.41)	0.816	28.42 (0.44) (27.54–29.30)	0.103	11.75 (0.23) (11.29–12.21)	0.314
	No	82.13 (0.40) (81.35–82.91)		33.27 (0.23) (32.82–33.71)		16.82 (0.16) (16.50–17.14)		12.95 (0.10) (12.75–13.15)		27.82 (0.17) (27.48–28.15)		12.06 (0.11) (11.84–12.28)	
Co-living	Ascendant relatives	82.51 (0.45) (81.63–83.39)	0.793	33.59 (0.24) (33.12–34.07)	0.165	16.80 (0.18) (16.43–17.13)	0.185	13.01 (0.12) (12.78–13.24)	0.427	28.02 (0.18) (27.65–28.38)	0.303	12.03 (0.12) (11.79–12.27)	0.071
	Friends/Flatmates	81.44 (0.80) (79.87–83.01)		32.46 (0.47) (31.54–33.39)		17.25 (0.29) (16.67–17.82)		12.71 (0.19) (12.34–13.09)		827.46 (0.33) (26.79–28.12)		11.84 (0.21) (11.42–12.26)	
	Descendent relatives	82.19 (1.95) (78.13–86.26)		34.62 (0.97) (32.60–36.64)		15.62 (0.81) (13.93–17.31)		12.34 (0.32) (12.34–13.66)		28.48 (0.78) (26.86–30.10)		11.33 (0.58) (10.13–12.54)	
	Alone	82.67 (1.71) (79.22–86.12)		33.09 (1.06) (30.96–35.22)		16.88 (0.59) (15.69–18.08)		13.07 (0.40) (12.27–13.87)		27.88 (0.81) (26.25–29.52)		12.67 (0.34) (12.00–13.35)	

Asterisks indicate significant differences (*p* < 0.05) (Mann–Whitney test when comparing a quantitative variable in two categories and Kruskal–Wallis test when comparing in three or more categories).
